# Barriers and Facilitators to Pre-Exposure Prophylaxis by Men Who Have Sex with Men and Community Stakeholders in Malaysia

**DOI:** 10.3390/ijerph20095669

**Published:** 2023-04-27

**Authors:** Aviana O. Rosen, Jeffrey A. Wickersham, Frederick L. Altice, Antoine Khati, Iskandar Azwa, Vincent Tee, Alma Jeri-Wahrhaftig, Jeffrey Ralph Luces, Zhao Ni, Adeeba Kamarulzaman, Rumana Saifi, Roman Shrestha

**Affiliations:** 1 Department of Allied Health Sciences, University of Connecticut, Storrs, CT 06269, USA; 2 Department of Internal Medicine, Section of Infectious Diseases, Yale School of Medicine, New Haven, CT 06510, USA; 3 Centre of Excellence for Research in AIDS (CERiA), Faculty of Medicine, University of Malaya, Kuala Lumpur 50603, Malaysia; 4 Infectious Diseases Unit, Department of Medicine, Faculty of Medicine, University of Malaya, Kuala Lumpur 50603, Malaysia; 5 Master of Health Research Ethics (MOHRE), Faculty of Medicine, University of Malaya, Kuala Lumpur 50603, Malaysia; 6 Yale School of Nursing, Yale University, Orange, CT 06477, USA

**Keywords:** HIV, men who have sex with men, pre-exposure prophylaxis, nominal group technique, patient preferences, Malaysia

## Abstract

Background: Men who have sex with men (MSM) in Malaysia are disproportionately affected by HIV. Pre-exposure prophylaxis (PrEP) is an evidence-based HIV prevention strategy; yet, uptake remains low among Malaysian MSM, who have a limited understanding of barriers to PrEP. Methods: We employed the nominal group technique (NGT), a structured mixed-methods strategy to understand the barriers and facilitators to PrEP use among Malaysian MSM, combined with a qualitative focus group. Six virtual focus group sessions, three among MSM (*n* = 20) and three among stakeholders (*n* = 16), were conducted using a video-conferencing platform. Rank-ordering of barriers from NGT was recorded, and thematic analysis was conducted for content. Results: Similar barriers were reported by MSM and community stakeholders, with aggregated costs associated with PrEP care (e.g., consultation with a clinician, medication, laboratory testing) being the greatest barrier, followed by limited knowledge and awareness of PrEP. Additionally, the lack of access to PrEP providers, the complex clinical protocol for PrEP initiation and follow-up, and social stigma undermined PrEP delivery. Qualitative discussions identified potential new strategies to overcome these barriers, including expanded outreach efforts to reach hard-to-reach MSM, a ‘one-stop’ delivery model for PrEP services, a patient-centered decision aid to guide PrEP uptake, and easy access to LGBT-friendly PrEP providers. Conclusion: Current barriers may be overcome through governmental subsidy for PrEP and evidence-informed shared decision aids to support both MSM and PrEP providers.

## 1. Introduction

Men who have sex with men (MSM) continue to bear a disproportionate burden of HIV in Malaysia. In 2021, MSM accounted for 63% of new HIV diagnoses in the country, a proportion that has been increasing over the past decade [1]. Numerous factors have been identified as the primary drivers fueling the HIV epidemic among MSM, including condomless sex, multiple sex partners, substance use (particularly amphetamine-type stimulants; ATS), and co-morbidities (e.g., depression, anxiety) [2,3,4,5]. In Malaysia, where same-sex practices are criminalized and highly stigmatized, reaching MSM and delivering HIV prevention remains challenging. Consequently, innovations are needed to guide one of the most effective HIV prevention strategies—pre-exposure prophylaxis (PrEP).

While PrEP is recommended as one of the most effective HIV prevention strategies by international agencies [6,7] and Malaysia’s Ministry of Health [8], the HIV prevention gap remains high in Malaysia due to suboptimal PrEP implementation. This prevention gap is heightened by findings suggesting a high level of awareness (80%) about and willingness (83.3%) to take PrEP [9]. Yet PrEP uptake among Malaysian MSM remains suboptimal, with only 18% of those at substantial risk of HIV infection actively taking PrEP [9].

To scale up PrEP beyond current levels, PrEP delivery must be guided by understanding potential barriers to delivery linked to opportunities. Though barriers and facilitators of PrEP implementation have been reported across various settings [10,11,12], it is crucial to rapidly assess barriers in the local context, as most of these barriers were reported from high-income countries. The challenges in Malaysia are further amplified due to being an LMIC, where stigma is heightened by documented stigma and discrimination against MSM, including from medical professionals [13,14,15,16]. Therefore, in this study, we used the nominal group technique (NGT) [17] in focus group sessions to rapidly assess barriers and facilitators to PrEP uptake by MSM and community stakeholders involved in PrEP delivery in Malaysia. This mixed-methods approach can complement previous studies that have used the quantitative method to assess willingness to use PrEP among Malaysian MSM [18,19], and thus provide contextual insights on these topics that are still not well understood but are vital for PrEP scale-up in the region.

## 2. Methods

### 2.1. Participants and Settings

Between March and May 2021, we recruited 36 participants (20 MSM and 16 community stakeholders). MSM were recruited through LGBT-friendly non-government organizations (NGOs) and social networking apps (e.g., Grindr, Hornet). Eligibility criteria included: (a) being 18 years or older; (b) being self-identified as MSM; and (c) understanding English or Bahasa Malaysia. Community stakeholders were health service providers who provided HIV-related services to the target population, including doctors, nurses, pharmacists, mental health counselors, community outreach workers, and NGO staff. Stakeholders were recruited through local NGOs, clinics, and hospitals.

### 2.2. Procedures

Participants were asked to complete a brief demographic survey prior to the focus group sessions. Each participant provided verbal consent before starting the session and was compensated for their participation (RM 40 for MSM and RM 80 for stakeholders; US $1~RM 4). The study protocol was approved by the Institutional Review Boards at the University of Connecticut and the University of Malaya.

Six virtual focus group sessions (three among MSM and three among stakeholders) were conducted using a video-conferencing platform, and each session lasted about 90 min. To ensure homogeneity, sessions were stratified based on PrEP use (MSM) and type of institutional affiliation (stakeholders). The first session with clients included those who had never used PrEP (i.e., PrEP naïve), whereas the second session included those on PrEP or who had previously used PrEP. The third session contained a mix of participants who had never been on PrEP and who had. The first session with community stakeholders included health service providers from a private LGBT-friendly clinic, the second included those from the University-affiliated hospital, and the third included individuals from an NGO-based LGBT-friendly clinic. On average, 6 participants (range: 4 to 9) were included in each session. A trained facilitator led the session, while a co-facilitator took notes, recorded non-verbal cues, and typed responses on a virtual whiteboard. Each session was audio-recorded and transcribed.

For five of the six focus groups, we employed the nominal group technique (NGT), a structured mixed-methods strategy of focus group discussion [17], to understand the barriers and facilitators to PrEP uptake among Malaysian MSM. The NGT process includes: (a) silent generation of responses; (b) round-robin feedback from participants to record ideas; (c) discussion of each recorded idea for clarification and evaluation; and (d) individual voting on priority ideas with the group decision being mathematically derived through rank-ordering [20]. We chose NGT because it creates quantitative estimates (rank-ordering) combined with in-depth qualitative information that can be implemented relatively quickly. The advantage of NGT compared to traditional focus groups is inclusivity, as it facilitates the equal participation of all group members despite power imbalances. NGT has also been widely successful in health-related research pertaining to identifying barriers and solutions to delivery of various health services [20,21,22]. One focus group session among MSM did not employ NGT, as saturation had been reached from the first two sessions but was still held as a semi-structured focus group session to identify key concepts pertaining to PrEP access among MSM.

Focus group questions were based on prior literature and then consolidated by consulting experts in HIV prevention and NGT methodology. The following question was the primary focus of each NGT session: “What kinds of things might get in the way of starting PrEP among men who have sex with men in Malaysia?” After participants were presented with this question, they silently generated unique ideas, either in writing or quietly among themselves. Then, using the ‘round-robin’ elicitation process, each person contributed a single idea recorded visually on a whiteboard. Additional rounds were completed until responses were saturated. Participants then engaged in a group discussion to clarify and evaluate ideas, and items were grouped by consensus and duplicate items removed. Next, individuals voted to prioritize items. Each participant could allocate up to three votes to cast on the listed item(s) they deemed most important in any chosen combination (e.g., three votes on one item, one vote per each of three items, etc.). Votes were immediately tallied and ranked (based on the total number of votes). The facilitator led a final discussion to review the participants’ results and ensure that they had face validity among participants. Participants were then asked to share potential solutions or facilitators to increase access to and utilization of PrEP among MSM in Malaysia based on the barriers identified.

### 2.3. Analytic Plan

All generated responses to questions were recorded, and votes were tabulated per item per group (i.e., MSM and stakeholders). We identified the highest-ranking responses per question, pooled them across groups, and organized them by question. Post-hoc analysis of the audio recordings and detailed notes collected during the NGT sessions were used to establish the major themes presented. Following recommendations for analyzing NGT session data across multiple groups, we consolidated raw ranking data and then conducted iterative rounds of thematic coding of responses and transcripts [23]. Using the top-ranked items from each session, two authors, AOR and AK, synthesized the results across sessions and tallied votes accordingly. Transcripts from the group sessions were reviewed and coded by AOR and were analyzed using thematic analysis. Responses from each session and qualitative data gathered during the group discussions were then analyzed. The results for the identified items were recorded to arrive at a final score for the prioritization process. The thematic framework categorized the highest-ranking responses, and the transcripts contextualized these priorities [24].

## 3. Results

### 3.1. Participant Characteristics

Among MSM, half (50%, *n* = 10) were Chinese, 40% (*n* = 8) were Malay, one was Indian (5%), and one was mixed ethnicity (5%). The majority (75%, *n* = 15) reported being sexually active in the last 6 months, although only 10% (*n* = 2) reported using condoms all the time. Most participants reported having previously been tested for HIV, with 85% (*n* = 17) having been tested in the last six months. The majority of the MSM sample (80%, *n* = 16) had heard of PrEP, 12 (60%) had ever taken PrEP, and 8 (40%) were currently on PrEP (Table 1).

Of the 16 community stakeholders, the majority (50%, *n* = 8) were Chinese. Most of them were medical doctors (38%, *n* = 6), followed by registered nurses (19%, *n* = 3) and administrators (19%, *n* = 3). These stakeholders worked at community-based organizations (33%, *n* = 5), private clinics (33%, *n* = 5), academic or university clinics (19%, *n* = 3), and government clinics (13%, *n* = 2).

### 3.2. Finding from MSM Sessions

#### 3.2.1. Barriers to PrEP Use

Table 2 presents barriers to PrEP use voted by MSM. Overall, the most common barrier included the high cost associated with PrEP services (9 votes), followed by limited knowledge and awareness of PrEP (7 votes). Three themes were equal, with six votes each: lack of access to PrEP providers, the complex clinical protocol for PrEP initiation and follow-up, and social stigma associated with PrEP use. Interestingly, PrEP naïve MSM voted for limited knowledge and awareness of PrEP, whereas those who had previously used PrEP voted for high cost as the top barrier.

Financial concerns related to PrEP care, including costs for consultation, laboratory testing, and PrEP medication, were expressed by MSM. Participants’ concern over high costs would decrease their willingness to pay and, eventually, affect their initiation or retention of PrEP care. “The cost of PrEP is relatively high, because they have to do it in a clinic and also have the test, the cost is going to be a bit pricy, especially for the youngsters, and also for the students because most of the MSM who are really sexually active are in a young stage.” (MSM session).

Another common barrier included a lack of knowledge and awareness about PrEP (e.g., how PrEP works, efficacy, potential side-effects, and misconception around PrEP) and where and how to access it. “For me, it’s almost the same about unaware about the PrEP itself. Because of me, there’s not much accessible information about what the side effects of this drug are and how easy it is to get the PrEP.” (MSM session).

Participants noted the difficulty navigating healthcare systems to access PrEP services. For example, one participant in an MSM session said, “Even if I tried googling, you don’t have that many choices that were to say this clinic offers this type of medication”. Participants also indicated a lack of access to trained providers where PrEP is routinely offered or who may not be sensitive to the LGBT community. “[Two issues] … finding PrEP providers and also unawareness about PrEP. First, if you go to the public clinics, still a lot of doctors and even doctors in Malaysia are unaware about the PrEP function. And secondly, if we want to find a private clinic outside Kuala Lumpur or Selangor [the state that includes Kuala Lumpur], it’s uh, it’s a tough process, especially in Sabah, Sarawak, and the northern part of Malaysia and east coast. And some of the public clinics, the doctors are so judgmental, and stigma about PrEP intake.” (MSM session).

Additionally, the complex and burdensome process of PrEP initiation (e.g., the need for multiple visits and intensive monitoring requirements) and frequent follow-up visits were discussed as barriers to PrEP use among MSM. “It takes a long time. For example, for one visit to the next visit, for the first test to the second HIV test is usually one month. So because of the need to travel, because certain people want to reduce the cost of the testing, they go to the government clinic to do the consultation because it’s free there. Then, after that, the doctor will give you a prescription, and then they need to go to a certain pharmacy to get the medication, but since the timeframe is quite long, sometimes they lose interest to do the PrEP.”

Participants presented social stigma from healthcare providers and community members (including other MSM) as an additional barrier to PrEP use. Many discussed being afraid that someone would assume they had HIV or would judge them for engaging in risky behaviors if they saw they were taking PrEP medication. One MSM stated, “The social anxiety that comes after that, like, I am HIV positive, kind of social perception that people will have at me.” Another expressed, “I think, for me, it will be fear of judgment, like I wanted to go, but then a fear of being judged by people there, yeah.”

MSM participants, particularly those who have already used PrEP, acknowledged that PrEP had to be used consistently to achieve optimal HIV preventive efficacy, which could be a concern for many MSM. Experience and/or anticipation of PrEP side effects and medication interactions were indicated as a potential barrier for participants to start using PrEP. “You know, you avoid medication if possible…I guess, um, I worry about potential side effects.” (MSM session).

#### 3.2.2. Facilitators of PrEP Use

MSM were further asked to discuss possible facilitators or solutions to the barriers to accessing PrEP services. The most common solutions included resolving the need for the government to subsidize costs associated with PrEP services, followed by the need to simplify the process of initiating PrEP. This included an expedited process to get a PrEP prescription (i.e., same-day PrEP, minimized repeat clinic visits, streamlined clinical procedures), thereby offering a more convenient solution to MSM and making the process less complex and demanding on the patient level. After describing the multi-step, burdensome, and time-consuming process to get on PrEP, one MSM participant stated, “It’s good if the government clinic can offer the consultation and provide the prescription on the same day, so that in one sitting, the patient or the client can get PrEP, instead of needing to wait for another one month.”

MSM participants further expressed the need for additional providers trained and willing to provide PrEP services to LGBT members and more modalities to access PrEP (e.g., telehealth, pharmacy, or key population-led model). “I guess just like, doctors talking about it more. Because I think when I have talked about it with doctors, it’s been brief. And it’s really like, ‘here’s an option’, but maybe more information from them could be helpful.”

### 3.3. Finding from Community Stakeholder Sessions

#### 3.3.1. Barriers to PrEP Use

Table 3 presents perceived barriers to PrEP use for MSM, as expressed from community stakeholders. Overall, the top voted barriers included high cost associated with PrEP services (17 votes), limited knowledge and awareness of PrEP among MSM (11 votes), lack of access to trained PrEP providers (10 votes), the complex clinical protocol for PrEP initiation and follow-up (6 votes), and low perception of HIV risk among MSM (3 votes). Interestingly, financial concerns associated with PrEP services were voted as the top barrier across all sessions; however, there were some differences in barriers noted among community stakeholders. For example, members from a private clinic indicated complex clinical protocol for PrEP initiation and follow-up as a second barrier. In contrast, those from NGO-based clinics and academic-led hospitals noted limited knowledge and awareness of PrEP among MSM and a lack of trained PrEP providers.

Barriers identified through the qualitative analysis (not part of the NGT process) included high pill burden (i.e., daily oral PrEP), logistical constraints accessing PrEP (e.g., long distance to PrEP provider, long wait for appointments, lack of transportation, need to miss multiple days of work to attend appointments), lack of coherent public health response and promotional activities, and social stigma associated with PrEP use. Community stakeholders also discussed how the COVID-19 pandemic exacerbated the structural barriers to accessing and staying on PrEP that already existed in Malaysia. For example, it was already difficult for MSM to take time off work to attend multiple appointments to initiate or maintain PrEP. Additionally, job loss during the pandemic has required many MSM to take up employment as daily contracted employees. Thus, taking a day off work meant not only losing an entire day’s pay, but also spending extra money on the travel needed to get to a clinic that provides PrEP, in addition to the costs associated with the HIV test and medication. From the community stakeholders’ perspective, they saw several clients discontinue PrEP use due to the exacerbated structural barriers during the pandemic. One stakeholder explained, “Things are slightly different now because of COVID-19. [MSM] have difficulty getting to the clinic, and sometimes the appointment keeps postponing, so I think that makes things a bit difficult for them to continue PrEP. One patient was actually stuck in Singapore, so I had to contact another doctor to try and get them to re-prescribe the medication for him and get a person in Malaysia to come and purchase the medication on his behalf and then send the PrEP to him in Singapore.”

#### 3.3.2. Facilitators of PrEP Use

The community stakeholders offered several solutions to address barriers to PrEP use among MSM, which included implementing novel ways to enhance PrEP education and encouraging the use of alternate PrEP regimes (e.g., on-demand PrEP, also known as PrEP 2-1-1 or event-driven PrEP). However, many providers are unaware of them and continue to offer only daily oral PrEP. “On-demand PrEP can be a viable solution for MSM who find it hard to adhere to taking a daily pill, who engage in intermittent risk behaviors, and for individuals who cannot afford daily PrEP.” (Stakeholder session).

To address the barriers around the lack of awareness about PrEP, community stakeholders discussed options for raising community awareness of PrEP. They expressed further concerns that even with community outreach efforts, it is usually only a specific group of mainly urban, knowledgeable, professional LGBT groups or “mainstream” MSM that receive these efforts; other at-risk groups of MSM are left out. A community stakeholder from Session 3 articulated the need to adjust outreach and language efforts to reach other groups of MSM, “…If you are really true to the whole description of what MSM is, I think there are many mainstream MSM, and then there are people who might not be English speaking, they are probably Malay speaking, they don’t mingle, they don’t have like a circle of gay friends. There is also Chinese-speaking MSM; there could also be working-class MSM, married men, men in smaller towns, so a lot of these groups of people have not really been reached out to or provided information about what PrEP is and why PrEP is something they can consider.” (Stakeholder session).

Furthermore, community stakeholders indicated the need to create seamless pathways for individuals to be linked to PrEP care from existing public health touchpoints (e.g., HIV/STI testing). They noted that the healthcare delivery system should empower MSM to better care for themselves regarding their sexual health and broaden their knowledge in this area, “You need to make an impression, you need to make sure they walk out the door feeling empowered, feeling that they actually know something after walking out of your clinic and sadly, this does not always happen.” (Stakeholder session). Nevertheless, not enough providers have the proper training to provide pre- and post-test counseling. As one stakeholder explained, “so they just jab you, take your blood, you go back, they will call you within 4–6 weeks when the results are ready”.

## 4. Discussion

Our findings provide a foundation for addressing the PrEP prevention gap among MSM in Malaysia, where MSM are highly stigmatized and discriminated against, including in healthcare settings [3,16]. Key barriers to PrEP uptake identified included financial concerns associated with PrEP care, lack of access to trained PrEP providers, complex processes for PrEP initiation, the social stigma associated with PrEP use, and limited knowledge and awareness of PrEP for both patients and providers. While there is limited research on PrEP use among Malaysian MSM [9,18,19], our findings are consistent with prior research exploring the barriers and facilitators to PrEP in general in other geographic settings [9,18,19,25].

We found financial concerns to be a primary barrier to PrEP use, which is consistent with prior studies that identified high costs of PrEP care (e.g., consultation visits, laboratory tests, PrEP medication) as a commonly reported reason for not initiating or discontinuing PrEP use [10,18,25]. Unlike in high-income countries, where costs related to PrEP care services are covered by insurance or financial assistance programs with no cost-sharing [26,27], it is not included in Malaysia’s national formulary of subsidized medications for HIV prevention. Since the conduct of this study, the government has agreed to make PrEP available for free at select public health clinics in high HIV prevalence settings [28]. However, the barriers to accessing this care may remain until such providers can deliver services in a non-judgmental manner, as healthcare providers often show strong negative bias towards key affected populations like MSM [13,14].

It is essential to implement strategies at the policy level to increase the affordability of PrEP medication, associated PrEP clinic visits, and laboratory monitoring, perhaps more broadly, beyond primary care clinics. This is especially true as governmental clinics continue with burdensome screenings and assessment algorithms that do not provide these on demand, as in some private settings. Particular attention should be paid to the needs and preferences of populations with the provision for client-centered differential models of PrEP delivery [7,29]. It is important for future efforts to incorporate decision aid for the provision of PrEP services (e.g., to navigate PrEP programs across various organizations, service capacity, cost, and wait times) that help individuals make informed decisions in accessing PrEP services [30,31].

Knowledge and awareness of PrEP among MSM and healthcare providers are crucial for PrEP scale-up. Consistent with prior studies in Malaysia and elsewhere [32,33,34], the lack of PrEP awareness and knowledge among patients and providers was cited as one of the most common reasons for suboptimal PrEP uptake. Specific knowledge about PrEP medication—mainly, how it works, its efficacy, and its potential side effects—remains a common gap among patients [19]. Positively framed messages detailing the empowerment inherent in PrEP as a highly efficacious and acceptable prevention method should be promoted through campaigns, clinicians, and health officials [35,36]. Among healthcare professionals, the disparity in knowledge is evident across the specialty. For example, existing literature indicates that infectious disease specialists are more knowledgeable about PrEP than other providers (e.g., family medicine, obstetrics, and pediatric practitioners). Strategies to increase awareness about PrEP, such as marketing campaigns with empowering messages tailored to highly impacted and stigmatized communities, are essential to educate individuals and link them to HIV prevention services. Additionally, improved education or training for clinicians across all specialties to increase PrEP knowledge and to alleviate concerns regarding PrEP safety and risk compensation are essential to overcome a range of barriers (e.g., lack of PrEP providers, lack of awareness) at the provider level [37]. Additionally, training to improve cultural humility would be important given that MSM experience stigma and discrimination in healthcare settings, which is identified as a barrier to PrEP use [10,18].

Many clinical settings require multiple in-person PrEP consult visits and laboratory tests for PrEP users. The complexity of the processes involved, particularly with long waits between visits and differences in the PrEP delivery model across various healthcare facilities (e.g., public vs. private), deters many potential PrEP users, as suggested in our study [38,39]. Additional barriers, such as fear of stigma, judgmental interactions, the lack of an LGBT-friendly setting, service delivery at inconvenient locations or times, a high pill burden, and not knowing where to get PrEP, further impact PrEP uptake and retention in care. Efforts to scale up PrEP can be facilitated by implementing and evaluating processes that streamline clinical PrEP initiation procedures for individuals without obvious contraindications, such as offering same-day PrEP initiation [40,41]. Furthermore, PrEP can be made more accessible through alternative provision strategies, including providing various modalities of PrEP (e.g., daily oral, on-demand, injectable PrEP), walk-in visits, telehealth/mHealth with home-based testing and mail-in PrEP orders, as well as a peer-driven model and PrEP services in non-traditional settings (e.g., community-based mobile clinics, pharmacies, mental health clinics, substance use clinics, emergency rooms) [42,43,44]. An additional promising option for improving PrEP scale-up includes a “one-stop” delivery model, which integrates and delivers all necessary services (e.g., mental health, substance use) at a single touchpoint, thus providing holistic care “under the same roof [45]. Such touchpoints could be a brick-and-mortar clinic or even delivered in the virtual space through smartphone apps.

Our varied findings across MSM sessions point to the differential need between MSM who have not been on PrEP or are PrEP naïve compared to those who have already used or are currently using PrEP. PrEP naïve MSM identified prominent barriers related to lacking information about PrEP and needing assistance initiating PrEP, whereas prior PrEP users identified barriers related to care and maintenance. These discrepancies suggest that tailored interventions based on PrEP status could be necessary and beneficial in increasing PrEP use and adherence among MSM, thus reinforcing the need for differentiated PrEP delivery for this at-risk group [46]. For example, future PrEP interventions for MSM who have never been on PrEP should focus on relaying information about PrEP (e.g., what it is, side effects, how to take it, and risk factors) and potentially helping MSM decide if PrEP is right for them. Efforts for MSM who had previously been on PrEP should focus more on the structural barriers, including ensuring participant privacy and confidentiality, simplifying the process of initiating PrEP and follow-up visits, and making PrEP more accessible.

Furthermore, our findings suggest an urgent need for tailored outreach activities and programs to reach these vulnerable subgroups. Community stakeholders mentioned that there are outreach efforts, but they unfortunately only reach the same groups of “mainstream” MSM that attend LGBT events, whereas rural MSM, MSM who use drugs, discreet MSM who may be married in heterosexual relationships, and others are all difficult to reach for HIV prevention. Future action-oriented research and prevention efforts should focus on enhancing outreach efforts to these other subpopulations of MSM, either through social media campaigns or mobile technologies, which have been accepted and efficacious in engaging vulnerable risk populations in HIV testing and PrEP on a global scale [47]. Alternatively, engaging MSM through online HIV self-testing programs is another way to reduce stigma and provide access to PrEP [48]. Peer navigators are another possible mechanism to increase PrEP literacy and to facilitate health system navigation using trusted peers [49].

Despite the many useful findings, there are limitations. As democratic as NGT is designed to be, certain stakeholders may be missing from the discussion, including those outside Greater Kuala Lumpur’s urban setting. Similarly, we may be missing the input from more stigmatized MSM who would not participate in MSM-related research. However, as NGT requires the participation of all present, non-participation is less of a concern, though some participants did not cast all three of their votes in each session. Finally, our focus group discussions only addressed barriers and facilitators to PrEP in daily pill form and cannot be generalized to other modalities of PrEP, such as injectable PrEP, which has recently been approved for HIV prevention. Despite these limitations, there are limited data on barriers to PrEP among MSM in Malaysia, which are included here.

## 5. Conclusions

PrEP is critical in reducing new HIV infections and ending the global HIV epidemic. Therefore, there is an urgent need to improve PrEP uptake among MSM, particularly in Malaysia, given the disproportionate impact of new HIV infections among MSM. Our data provide evidence of the most prominent barriers and facilitators to PrEP among MSM in Malaysia, identified by MSM and community stakeholders in Malaysia. The reasons for low PrEP uptake among Malaysian MSM are complex, and the approaches to increasing PrEP uptake among MSM in Malaysia will require a multisectoral response, including an empowering public health marketing strategy, innovations in clinical approaches, novel ways to access PrEP (e.g., telehealth, pharmacy, community-based, peer-driven), streamlining clinical procedures (e.g., same-day PrEP initiation, alternative PrEP regimens, minimized repeat clinic visits/labs), and national policies supportive of access to PrEP services for those in most need.

## Figures and Tables

**Table 1:** Participant characteristics.

MSM (*n* = 20)		Community Stakeholders (*n* = 16) *	
Variable	*n* (%)	Variable	*n* (%)
Ethnicity		Ethnicity	
Chinese	10 (50%)	Chinese	8 (50%)
Malay	8 (40%)	Malay	3 (19%)
Indian	1 (5%)	Indian	3 (19%)
Mixed	1 (5%)	Native Sabahan	1 (6%)
Sexual Orientation		Occupation	
Gay/PLU	20 (100%)	Outreach Worker	1 (6%)
Sexual Activity (Past 6 months)		Administrator	3 (19%)
Yes	15 (75%)	Medical Doctor	6 (38%)
No	5 (25%)	Registered Nurse	3 (19%)
Condom Use (Past 6 months)		Pharmacist	1 (6%)
Never	2 (10%)	Psychologist	1 (6%)
Sometimes	2 (10%)	Type of Health Facility	
Most of the time	9 (45%)	Community-based Organization	5 (33%)
All the time	2 (10%)	Clinic/Hospital: Private	5 (33%)
No Response	5 (25%)	Clinic/Hospital: Academic	3 (19%)
Substance Use (Past 6 months)		Clinic/Hospital: Government	2 (13%)
Alcohol	5 (25%)	Facility offers HIV Testing	
Cigarettes	2 (10%)	Yes	14 (88%)
Crystal Meth	3 (15%)	No	1 (6%)
GHB/GBL	1 (5%)	Involved in providing PrEP services	
Poppers	2 (10%)	Yes	11 (69%)
Marijuana	1 (5%)	No	4 (25%)
None	10 (50%)	Number of MSM patients on PrEP	
Last HIV test		0–10	4 (25%)
12+ months ago	1 (5%)	11–50	9 (56%)
7–9 months ago	1 (5%)	51–100	0 (0%)
4–6 months ago	8 (40%)	100	2 (13%)
<3 months ago	9 (45%)		
Never	1 (5%)		
HIV Status			
Negative	19 (95%)		
Unknown	1 (5%)		
Ever heard of PrEP			
Yes	16 (80%)		
No	4 (20%)		
Ever taken PrEP			
Yes	12 (60%)		
No	8 (40%)		
Currently on PrEP			
Yes	8 (40%)		
No	12 (60%)		

* Data available for 15 participants only.

**Table 2:** Key barriers to accessing PrEP reported by MSM in Malaysia.

Identified Barriers to PrEP	Voting Results *		
	PrEP Naïve (*n* = 9)	On PrEP (*n* = 6)	Total (*n* = 15)
High cost associated with PrEP services **	4	5	9
Limited knowledge and awareness of PrEP among MSM	7	-	7
Lack of access to trained PrEP providers	2	4	6
Complex clinical protocol for PrEP initiation and follow-up	2	4	6
Social stigma associated with PrEP use	2	4	6
Concerns about side effects and medication interactions	3	-	3
Need decision aid to guide PrEP decision-making	3	-	3

* Participants received 3 votes in each session to allocate across the top 3 most important barriers. They could apply 2+ votes to one barrier if they felt it was needed. ** Includes cost for the pills, initiation tests and appointments, follow-up tests and appointments.

**Table 3:** Key barriers to accessing PrEP as identified by community stakeholders in Malaysia.

Identified Barriers to PrEP	Voting Results *			
	Private Clinic (*n* = 4)	University Hospital (*n* = 6)	LGBT-Friendly NGO (*n* = 6)	Total (*n* = 16)
High cost associated with PrEP services	5	8	7	17
Limited knowledge and awareness of PrEP among MSM	2	4	7	11
Lack of access to trained PrEP providers	1	5	4	10
Complex clinical protocol for PrEP initiation and follow-up	4	2	-	6
Low perception of HIV risk among MSM	3	-	-	3
Social stigma associated with PrEP use	0	0	-	0

* Participants received 3 votes in each session to allocate across the top 3 most important barriers. They could apply 2+ votes to one barrier if they felt it was needed. 0 represents barriers that were identified in the NGT process but received no votes—signifies lower priority than the other highly voted barriers.

## Data Availability

The data presented in this study are available on request from the corresponding author.
